# Equiprobable discrete models of site-specific substitution rates underestimate the extent of rate variability

**DOI:** 10.1371/journal.pone.0229493

**Published:** 2020-03-02

**Authors:** Frank Mannino, Sadie Wisotsky, Sergei L. Kosakovsky Pond, Spencer V. Muse

**Affiliations:** 1 Bioinformatics Research Center, North Carolina State University, Raleigh, NC, United States of America; 2 Institute for Genomics and Evolutionary Medicine, Temple University, Philadelphia, PA, United States of America; 3 Department of Statistics, North Carolina State University, Raleigh, NC, United States of America; SOKENDAI (The Graduate University for Advanced Studies), JAPAN

## Abstract

It is standard practice to model site-to-site variability of substitution rates by discretizing a continuous distribution into a small number, *K*, of equiprobable rate categories. We demonstrate that the variance of this discretized distribution has an upper bound determined solely by the choice of *K* and the mean of the distribution. This bound can introduce biases into statistical inference, especially when estimating parameters governing site-to-site variability of substitution rates. Applications to two large collections of sequence alignments demonstrate that this upper bound is often reached in analyses of real data. When parameter estimation is of primary interest, additional rate categories or more flexible modeling methods should be considered.

## Introduction

The inclusion of site-to-site rate variability in evolutionary models for DNA sequences better reflects underlying biology and has been shown to almost universally improve model fit. Therefore, the use of such models has become commonplace. The earliest practical models for allowing nucleotide substitution rates to vary across sites [1] assumed a gamma distribution of rates controlled by a single shape parameter, *α*. Methods incorporating the continuous gamma distribution proved to be too computationally expensive when applied to larger data sets, and this fact led to the development of methods based on discretizing the continuous distribution into a small number, *K*, of equiprobable rate categories [2]. This “discrete gamma” approach proved to be an effective compromise of biological realism and computational complexity, and it has evolved into the *de facto* standard technique for modeling the evolution of molecular sequences. Yang [2, 3] showed that in terms of measures of model fit, there is typically little to be gained by using more than a few rate categories. Coupled with the fact that the computational expense increases linearly as additional categories are added, the community rather quickly converged on choosing *K* = 3 to 5 categories. While the computational cost of choosing larger values of *K* was once significant, that is no longer the case for most data sets- even very large ones. In this work we demonstrate that while there may be limited value for using more rate categories in terms of model fit, adding these additional categories offers important improvements in terms of statistical bias and robustness when a primary goal is to actually estimate the parameter values of the underlying rate distributions. Specifically, we demonstrate that the choice of *K* places a mathematical upper bound on the variance of the rate distribution when standard discrete gamma techniques are employed. This bound has important consequences when we estimate quantities such as the coefficients of variation of synonymous and nonsynonymous substitution rates. Using two large collections of data sets we show empirically that this bias is not simply of theoretical concern, but can have substantive practical impacts on our analyses and inferences.

## Results and discussion

As noted above, the most common approach for modeling site-to-site variability of substitution rates is by using a discretized version of a continuous probability distribution, most often the gamma. We begin this section by proving a general mathematical result showing that the discretization process imposes an upper bound on the variance of the distribution. This result applies not only to the case of the discretized gamma, but to any discrete distribution with equiprobable rates.

Consider a non-negative discrete-valued random variable *X* with mean *μ* and *K* equiprobable possible values 0 ≤ *X*_1_ ≤ *X*_2_ … ≤ *X*_*K*_, where Pr{*X* = *X*_*i*_} = 1/*K*. It follows that

$$\begin{array}{c} \begin{array}{ccc} \text{Var}(X) & := & E(X^{2})-\mu^{2}\\ & = & \frac{1}{K}\sum_{i=1}^{K}X_{i}^{2}-\mu^{2}\\ & \leq & \frac{1}{K}{\left(\sum_{i=1}^{K}X_{i}\right)}^{2}-\mu^{2}\\ & = & K{\left(\sum_{i=1}^{K}X_{i}/K\right)}^{2}-\mu^{2}\\ & = & \left(K-1\right)\mu^{2}. \end{array} \end{array}$$

The inequality of the third line holds because all *X*_*i*_ are non-negative. Indeed, all higher moments of the discrete distribution are subject to similar bounds:

$$\begin{array}{c} \begin{array}{ccc} \text{E}(X^{n}) & := & \frac{1}{K}\sum_{i=1}^{K}X_{i}^{n}\\ & \leq & \frac{1}{K}{\left(\sum_{i=1}^{K}X_{i}\right)}^{n}\\ & = & K^{n-1}{\left(\sum_{i=1}^{K}X_{i}/K\right)}^{n}\\ & = & K^{n-1}\mu^{n}. \end{array} \end{array}$$

Consequently, if the true variance of a continuous distribution exceeds the upper bound imposed by choice of *K*, estimates derived from the discretized version of that distribution will necessarily be negatively biased. The coefficient of variation (CV) of *X*, defined as $\sqrt{\text{Var}(X)}/E\left(X\right)$, is an important quantity in studies of rate variation and the neutral theory (*cf* [4]). Application of the variance result above demonstrates that its estimates are bounded above by $\sqrt{K-1}$ if derived using standard equiprobable discretization techniques.

The most common model for site-to-site rate variation is the gamma distribution with unit mean and shape parameter *α*, and the estimate of *α* is typically used to quantify the amount of site-to-site variability. One can estimate the variance (or CV) of this distribution in several ways. Some quantities, such as variance, are simple functions of *α*. If they are derived by plugging an estimate of *α* into the desired function, the upper bound bias problem is largely avoided. However, using such a plug-in estimate implies that we assume the continuous gamma distribution accurately describes the distribution of rates across sites. In addition to those robustness issues, there is also evidence that the *α* estimate itself is negatively biased [5, 6]. The more common approach, and the one we use in this work, estimates these quantities directly from the discretized distribution (i.e., directly from the *X*_*i*_) and can lead to significant bias. Such a situation arises whenever there is no simple functional relationship between *α* and the quantity of interest, or when one prefers not to rely on a fully parametric description of the rate distribution. One common application would be empirical Bayes analysis to infer rates at individual sites [7].

The technique of discretizing a continuous distribution offers substantial computational advantages over modeling approaches using the full continuous distribution, and it adds only a small number of parameters to be estimated (the single shape parameter *α* in the case of the gamma). However, this parametric choice also imposes restrictions on the overall shape of the rate distribution. For instance, it eliminates the possibility of bi-modal or U-shaped distributions. It is possible— but rarely implemented— to avoid this by using a general discrete distribution (GDD, [7, 8]). In such an approach the user chooses a number of categories of rates for their data, and then the values of those rates and their frequencies are estimated. Thus, with each additional category one adds two parameters to be estimated– the rate itself, and the fraction of sites evolving with that rate. In what follows we also consider the performance of this GDD approach for parameter estimation, in order to highlight the underappreciated estimation bias of certain distributional quantities that is the result of using today’s “default” models of rate variation.

**Fig 1:**
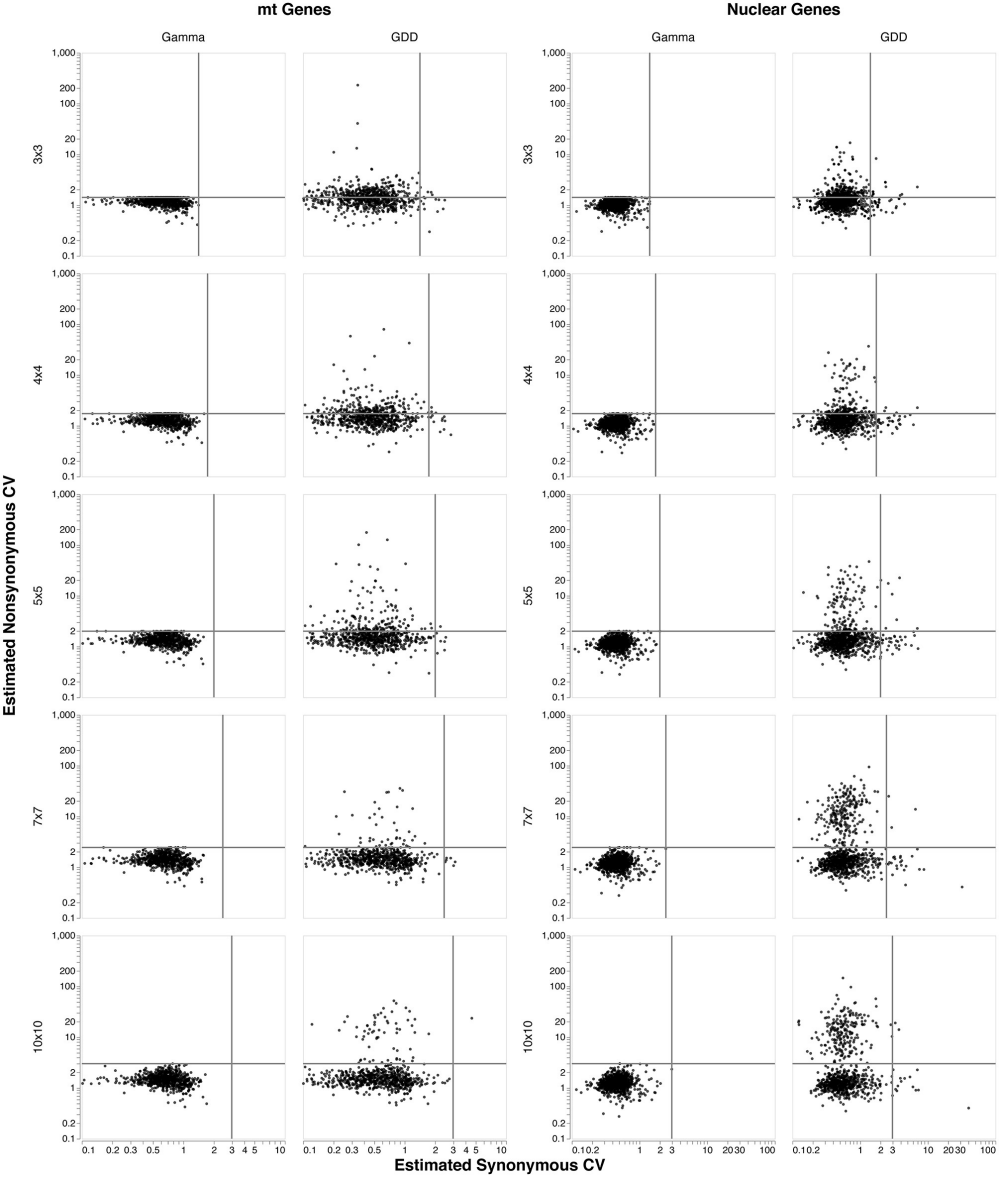
Impact of the number of rate categories and choice of estimation procedure on estimates of CV. For each of 13 mtDNA genes for 56 metazoan orders (721 total alignments, left plots), and for 1, 000 nuclear gene alignments (right plots) from the Selectome database, the estimated coefficients of variation (CV) for nonsynonymous rates (vertical axis) and synonymous rates (horizontal axis) are plotted for an increasing number of rate categories, *K* = 3, 4, 5, 7, 10. The plots in the left half of the figure show estimates obtained using the standard discrete gamma approach, while those in the right half were found using the GDD. The solid lines in each subplot mark the maximum possible CV estimate for the discrete gamma approach, $\sqrt{K-1}$. Observe that as the number of rate categories increases, fewer estimates reach the upper bound for the discrete gamma; estimates for the GDD have no such constraint, regardless of the number of rate categories. Please note that the axes do not use a linear scale.

In Fig 1 we plot the estimated CVs of synonymous (horizontal axis) and nonsynonymous (vertical axis) substitution rates obtained using both the discrete gamma and the GDD procedures. These estimates come from collections of 721 mitochondrial gene alignments (left) and 1, 000 nuclear gene alignments (right). For the discrete gamma approach, the discretization-induced upper bound is reached for many alignments, primarily when estimating the CV of the nonsynonymous rate distribution. As the number of categories *K* increases from 3 to 10, progressively fewer alignments yield estimates that reach the upper bound: 13.7% for *K* = 3, but only one for *K* = 10 in the mtDNA genes. For the nuclear genes those values are 4.1% for *K* = 3 and none for *K* = 10. (In all cases a handful of estimates fall very close to the upper bound without actually reaching it).

While increasing the number of categories greatly reduces the number of data sets reaching the upper bound, use of the GDD for estimation completely eliminates this problem. It is immediately evident upon comparing the left (discrete gamma) and right (GDD) plots that use of the discrete gamma significantly reduces the range of both synonymous and nonsynonymous CV estimates, even when the upper bound is not reached. A reasonable response to the behavior seen in these plots is to suggest that one should always use GDD- it eliminates the upper bound issue, and even for 10 rate categories (where the upper bound is rarely reached) the distributions of discrete gamma CV estimates are visibly compressed relative to those in the GDD plots. This is not bad advice, but one must also take into consideration the impacts on computation time and on statistical variability. The computational burden of switching from discrete gamma to GDD is real, but typically not prohibitive with modern computing and datasets of typical size. In the analyses presented here, for example, shifting from a 4 × 4 discrete gamma approach to a 4 × 4 GDD resulted in about a 25% increase in computing time. Adding categories to the discrete gamma distribution is far more costly, with the move from 3 × 3 to 10 × 10 increasing compute times 8- to 10-fold. In terms of statistical variability, adding categories to the discrete gamma does not add any parameters to the model, since the additional categories are simply helping to provide a better approximation of the likelihood surface. When categories are added to the GDD analysis, however, two parameters (a rate and its frequency) are added with each additional category. Thus, one could rather quickly find a number of parameters in their model that are doing little more than adding unnecessary variability to estimates. On the positive side, the GDD plots reveal that a relatively small number of categories is typically sufficient to capture most of the range of the CV estimates.

To summarize, when one’s primary objective is to obtain accurate parameter estimates, the current standard of using the discrete gamma with a small number of rate categories is likely to yield negatively-biased estimates for many data sets as a result of the upper bound imposed by this approach. Increasing the number of categories is beneficial as it reduces the impact of the upper bound problem, and using the GDD with four or five categories is even more sound practice. If accurate estimation of parameters such as the CV is not central to the study, or if rate variation is a nuisance parameter— as is often the case in phylogenetic inference— then the standard discrete gamma approaches with four or five rate categories will likely suffice.

## Materials and methods

The results discussed above and displayed in Fig 1 are based on analyses of two large collections of data sets. From GenBank we compiled a set of complete mtDNA genome sequences representing 56 metazoan orders. We limited our collection to orders with at least 5 taxa present, and selected a phylogenetically representative set of 25 taxa for orders where larger numbers were available. This process yielded a total of 721 total mtDNA data sets. Since the nature of molecular evolution varies between nuclear and mitochondrial genomes, we also selected 1, 000 alignments from the Selectome database [9]. The mtDNA sequences were aligned at the gene level using the MUSCLE [10] algorithm as implemented in Mesquite v. 2.74 [11]. The individual gene alignments were then concatenated using FASconCAT [12], and these partitioned alignments were used to create trees with MrBayes v3.2.1 [13]. Trees for each gene within a genome alignment were forced to be the same, but each gene was given its own evolutionary parameters. The nuclear gene alignments and trees provided by Selectome were used without modification.

All DNA sequence alignments and trees used in this study are available for download at https://github.com/srwis/variancebound. Analyses of these data files were carried out with HyPhy [14], using the dNdSRateAnalysis batch file [15] available at https://github.com/veg/hyphy/. The discrete gamma estimates were obtained using that batch file’s option 1: [Syn:Gamma, Non-syn:Gamma]; the GDD estimates were found using option 3: [Independent Discrete].
